# Improving EDLC Device Performance Constructed from Plasticized Magnesium Ion Conducting Chitosan Based Polymer Electrolytes via Metal Complex Dispersion

**DOI:** 10.3390/membranes11040289

**Published:** 2021-04-14

**Authors:** Shujahadeen B. Aziz, Elham M. A. Dannoun, M. H. Hamsan, Rebar T. Abdulwahid, Kuldeep Mishra, Muaffaq M. Nofal, M. F. Z. Kadir

**Affiliations:** 1 Hameed Majid Advanced Polymeric Materials Research Lab., Physics, College of Science, University of Sulaimani, Qlyasan Street, Sulaimani 46001, Kurdistan Regional Government, Iraq; rebar.abdulwahid@univsul.edu.iq; 2 Department of Civil Engineering, College of Engineering, Komar University of Science and Technology, Sulaimani 46001, Kurdistan Regional Government, Iraq; 3 Associate Director of General Science Department, Woman Campus, Prince Sultan University, P.O. Box 66833, Riyadh 11586, Saudi Arabia; elhamdannoun1977@gmail.com; 4 Centre for Foundation Studies in Science, University of Malaya, Kuala Lumpur 50603, Malaysia; hafizhamsan93@gmail.com (M.H.H.); mfzkadir@um.edu.my (M.F.Z.K.); 5 Department of Physics, College of Education, University of Sulaimani, Old Campus, Sulaimani 46001, Iraq; 6 Department of Physics and Materials Science, Jaypee University, Anoopshahr 203390, India; kuldeep.mishra@mail.jaypeeu.ac.in; 7 Department of Mathematics and General Sciences, Prince Sultan University, P.O. Box 66833, Riyadh 11586, Saudi Arabia; muaffaqnofal@gmail.com

**Keywords:** plasticized polymer electrolyte, metal complex, structural analysis, impedance study, TNM and LSV analysis, CV, EDLC device

## Abstract

The current work shows the preparation of plasticized chitosan-magnesium acetate Mg(CH_3_COO)_2_-based polymer electrolyte dispersed with nickel (Ni) metal complexes via solution casting. Investigations of electrical and electrochemical properties of the prepared polymer composite electrolyte were carried out. The structural and optical properties of the samples were studied using X-ray diffraction (XRD) and UV-Vis spectroscopy techniques. The structural and optical outcomes revealed a clear enhancement in both absorbance and amorphous nature of the samples upon the addition of Ni metal complexes. Through the simulation of impedance data, various ion transport parameters were calculated. The electrochemical performance of the sample was examined by means of transference number measurement (TNM), linear sweep voltammetry (LSV) and cyclic voltammetry (CV) measurements. The TNM analysis confirmed the dominance of ions as the main charge carrier in the electrolyte with t_ion_ of (0.96) compared to only (0.04) for t_el_. The present electrolyte was stable in the range of 0 V to 2.4 V, which was obtained from linear sweep voltammetry (LSV). A result from CV proved that the electrical double-layer capacitor (EDLC) has a capacitive behavior as no redox peaks could be observed. The presence of Ni improved the charge–discharge cycle of the EDLC due to its amorphous behavior. The average performances of the EDLC were recorded as 41.7 F/g, 95%, 5.86 Wh/kg and 628 W/kg for specific capacitance, coulombic efficiency, energy and power densities, respectively. The fabricated EDLC device was found to be stable up to 1000 cycles.

## 1. Introduction

In times of intensive development of electric vehicles around the world, supercapacitors (SCs) play a large role [1]. SCs act as a power buffer during energy transfer from the fuel cell of the propulsion engine of the vehicle [1]. Lithium–ion batteries are usually used as the main energy source due to their high energy density, but when it comes to sudden demand for high power, e.g., acceleration or braking, high power density characteristic of SCs comes in handy [2]. Electrical double-layer capacitor (EDLC) is the type of SC that has the easiest fabrication method [3]. Many types of modified carbon with surface area and high porosity are used as the activating material in the EDLC electrode [4]. EDLCs are commonly used in electronic devices, communication gadgets, aviation and hybrid transportation [5].

Several research groups have focused on improving EDLC by developing new materials in an attempt to gain elevated electrochemical capacitance. The most popular and interesting materials are activated carbon aerogels, graphene and carbon nanofibers. Among these materials, activated carbon is relatively the best and most proper active material for constructing electrodes in EDLC devices that possess satisfactory electrical conduction, high specific surface area (2500 m^2^/g) and low cost [6]. To improve the electrical properties of the electrodes, carbon black is commonly inserted in the electrode composition. Carbon black is known as a para-crystalline material that has a high surface-area-to-volume ratio (25 to 1500 m^2^/g), which is relatively lower than that of activated carbon. It also acts as a reinforced filler for dimensional stability of the electrode materials [7].

The literature revealed that electrochemical stability of the electrolyte plays the key role in determining the overall performance of EDLC, particularly its life cycle and safety [4,8]. The electrochemical potential window is usually expressed in terms of the upper and lower ranges of oxidation and reduction reactions [4]. Despite the constituent of the electrolyte, both geometry and compatibility of the electrolyte with the electrodes also impact the electrochemical stability of the electrolyte [4,9]. Due to the existing trade-off between the electrochemical potential window and ionic conductivity, one should carefully tune the properties of the electrolyte in EDLC to achieve both high energy and power densities [9,10]. Since the EDLC device undergoes the charge–discharge process, a voltage difference generally accumulates on the used electrolyte that might reach a value to decompose the electrolyte and ultimately fail the device. Thus, in order to confirm the eligibility and suitability of an electrolyte for energy device applications, it is of special importance to determine the potential window using the linear sweep voltammetry (LSV) test. Previous works have shown that the polymer electrolytes with a potential window larger than 1 V can be viable for electrochemical device applications [11,12,13,14].

Magnesium salts comes with several interesting properties such as high reduction potential, safety, low equivalent weight and reasonable price [15]. Mg^2+^ is considered as large ions where it is beneficial in the conduction mechanism. A large ion has low attraction force with the polymer host compared to small ions. Despite all these unique characteristics, less attention has been paid to Mg^2+^-based polymer electrolytes (PEs) [16]. Hassan et al. [17] have claimed that the inclusion of magnesium salt enhanced the amorphousness of the biopolymer host and the interactions between Mg^2+^ and polymer were detected through Fourier-transform infrared spectroscopy (FTIR) analysis. Polu et al. [18] have shown that the glass transition temperature of polyvinyl alcohol (PVA) decreased with the presence of magnesium acetate (Mg(CH_3_COO)_2_) [18]. This is due to the weakening of the attractive force between polymer chains. It has been reported in [19] that the transport properties such as ionic mobility, diffusivity and number density of starch-based electrolyte depend on the amount of magnesium sulphate (MgSO_4_).

In earlier studies, it was emphasized that the amorphous phase of polar polymers can be improved using metal complexes [20]. Thereby, the DC ionic conductivity increases as the amorphous phase is dominating [20,21,22]. Overall, the metal complex inclusion into the polymer electrolytes can considerably increase the performance of the electrolyte for storage device commercialization [20]. Asnawi et al. [20] have shown that the addition of zinc metal complex into the chitosan (CS)-based electrolyte is aimed at improving the amorphous phase within the polymer body to be ionically satisfactory conduction material. The previous study [23] proved that the EDLC device has a constant stability up to 400 cycles. The energy concerns and related environmental issues have made the field of energy and particularly energy storage devices a very hot topic since the beginning of the 21st century. Every year, thousands of scientific works are published on energy storage devices. Through our researches in this field we are aiming toward commercializing polymer-based energy storage devices. Achieving this goal requires testing a variety of polymer-based electrolyte systems and tuning different properties of the polymer electrolyte in order to reach the optimum solution. Using biodegradable polymer-based electrolytes such as CS can have both environmental and economic benefits. However, the electrical, mechanical and physical properties of these natural polymers need significant alteration to best fit the energy device applications. In this regard, different approaches can be taken to achieve this goal, including using various fillers like metal complexes and plasticizers. The current work shows proof of the influence of the metal complex and plasticizer on the performance of the EDLC assembly up to 1000 cycles.

## 2. Experimental Detail

### 2.1. Materials and Electrolyte Synthesis

CS with moderately high molecular mass of approximately 310,000 to 375,000 g/mol and glycerol were used as raw materials in the preparation of the plasticized polymer. From Sigma-Aldrich (Kuala Lumpur, Malaysia), the other raw materials were received and used without further purification, including acetic acid and magnesium acetate. The procedure comprises dispersion of 1 g of CS in 50 mL of acetic acid (1 wt.%) solution followed by addition of 40 wt.% (0.666 g) of magnesium acetate (Mg(CH_3_COO)_2_) salt. Afterwards, the mixture was stirred continuously using a magnetic stirrer until complete homogeneous dispersion was gained at room temperature. To make plasticization, 42 wt.% of glycerol (1.206 g) was added into this homogenous dispersed mixture with continuous stirring until a clear solution was obtained. Subsequently, to the plasticized sample, (CS-glycerol- Mg(CH_3_COO)_2_) system, 10 mL of diluted Ni metal complex was added.

The methodology of preparation of metal-complex as a green approach has been documented in the earlier work [24]. To cast the final mixture, the solution was then spilled carefully into a number of clean and dry glass Petri dishes. Evaporation of the casted sample was performed by leaving the cast films at room temperature in order to dry.

### 2.2. X-ray Diffraction (XRD) and UV-Vis Measurements

The UV-Vis spectra of the samples were obtained through employing a double beam UV-Vis-NIR spectrophotometer (Model: Lambda 25), Perkin Elmer (Waltham, MA, USA) in the wavelength range from 180 to 1100 nm. 

The structural properties of the samples were investigated at room temperature using a D5000 X-ray diffractometer (Malvern Panalytical Ltd., Malvern, UK) working at 40 kV voltage and 45 mA current correspondingly. A monochromatic beam of X-ray was applied to the samples with wavelength (λ = 1.5406 Å) and glancing angles (2θ) between 10 and 80 with a 0.05 step size.

### 2.3. Impedance and Circuit Simulation

Hioki 3531-Z Hi Tester in the frequency range of 50 Hz to 1MHz was used in data collecting of impedance data points at room temperature. This was carried out by mounting the sample films on a conductive holder with 2 cm^2^ stainless steel electrodes.

Studying ion transport was performed to provide insight into electrical equivalent circuit (EEC) model, showing the whole picture of the system [25,26,27,28]. The EEC had two possible designs; a parallel combination of bulk resistance and constant phase element (CPE) and bulk capacitance (Z_CPE_) in series with another CPE from the tilted spike region response. The CPE was used in place of capacitor, reflecting depressed semicircle response [25]. To draw the EEC design, the obtained impedance data had to be fitted by simulation and the impedance of Z_CPE_ could be written as follows [27,28]:

(1) $$Z_{\mathrm{CPE}}=\frac{\cos(\pi n/2)}{Y_{m}\omega^{n}}-j\frac{\sin(\pi n/2)}{Y_{m}\omega^{n}}$$

where Y_m_ represents CPE capacitance, ω is the angular frequency and n is the factor as a measure of the deviation of the plot from vertical axis in the complex impedance plots. Herein, the values of Z_r_(real) and Z_i_(imaginary) can be mathematically represented and can be used in the equivalent circuit design:

(2) $$Z_{r}=R_{s}+\frac{R_{1}+R_{1}^{2}Y_{1}\omega^{n1}\cos({\pi n}_{1}/2)}{1+2R_{1}Y_{1}\omega^{n_{1}}\cos({\pi n}_{1}/2)+R_{1}^{2}Y_{1}^{2}\omega^{2n_{1}}}+\frac{\cos({\pi n}_{2}/2)}{Y_{2}\omega^{n_{2}}}$$

(3) $$Z_{i}=\frac{R_{1}^{2}Y_{1}\omega^{n_{1}}\sin({\pi n}_{1}/2)}{1+2R_{1}Y_{1}\omega^{n_{1}}\cos({\pi n}_{1}/2)+R_{1}^{2}Y_{1}^{2}\omega^{2n_{1}}}+\frac{\sin({\pi n}_{2}/2)}{Y_{2}\omega^{n_{2}}}$$

### 2.4. Transference Number Analysis

The cell polarization of stainless steel (SS) | conducting solid polymer electrolyte (SPE) | SS was used in the analysis of ion (t_ion_) and electron (t_el_) transference numbers (TNM). The working voltage was held constant at 0.8 V to perturb the electrolyte media. The V&A Instrument DP3003 digital DC power supply (V & A Instrument, Shanghai, China) was used to measure t_ion_ at room temperature using the following relationships:

(4) $$t_{\mathrm{ion}}=\frac{I_{i}-I_{\mathrm{ss}}}{I_{i}}$$

(5) $$t_{\mathrm{el}}=1-t_{\mathrm{ion}}$$

where current at the initial and steady state are symbolized as I_i_ and I_ss_, respectively.

### 2.5. Linear Sweep Voltammetry (LSV)

Potential stability of the PE was acquired from LSV. The cell arrangement for LSV analysis was SS | highest conducting SPE | SS. This analysis was achieved using Digi-IVY DY2300 potentiostat with scan rate 50 mV/s.

### 2.6. Fabrication of EDLC

Powdering the mixture of 81.25% activated carbon and 6.25% carbon black by grinding was carried out using a planetary ball miller. A solution of 12.5% of polyvinylidene fluoride (PVdF) was added to 15 mL N-methyl pyrrolidone (NMP) and stirred until complete dissolution was obtained. The powder mixture was then poured into the PVdF–NMP solution and then stirred until a thick black solidified gel form was obtained. Coating of the gel form film on an aluminum foil using a doctor blade was performed and then it was dried in an oven at 60 °C. To achieve full elimination of tiny moisture from the electrodes, a desiccator was used. The EDLC assembly consisted of two electrodes with geometric surface area of 2.01 cm^2^ that sandwiched a conducting electrolyte. The cell was packed in CR2032 coin and mounted in a Teflon case. 

### 2.7. Characterization of the EDLC

Cyclic voltammetry (CV) analysis was run at various scan rates (10 to 100 mV/s) using Digi-IVY DY2300 potentiostat (Neware, Shenzhen, China). This was to verify the influence of scan rate on specific capacitance (C_CV_) value using the following relationship:

(6) $$C_{\mathrm{CV}}=\int_{V_{i}}^{V_{f}}\frac{I\left(V\right)\mathrm{dV}}{2\mathrm{mv}\left(V_{f}-V_{i}\right)}$$

where V_i_ and V_f_ are 0 V and 0.9 V, respectively. The I(V)dV is the area under CV response and m is the weight of each electrode. In this study, the current density of 0.5 mA/cm^2^ was kept constant in the EDLC assembly during the experimental time scale. In the evaluation of EDLC assembly, several crucial parameters had to be taken into consideration, such as specific capacitance from charge–discharge (C_CD_), equivalent series resistance (ESR), energy (E) and power density (P), discovered using the following expressions:

(7) $$C_{\mathrm{CD}}=\frac{i}{\mathrm{sm}}$$

(8) $$\mathrm{ESR}=\frac{V_{d}}{i}$$

(9) $$E=\frac{C_{s}V^{2}}{2}$$

(10) $$P=\frac{V^{2}}{4m\left(\mathrm{ESR}\right)}$$

where s, i, V_d_ and V are gradient of the discharge response, current applied, drop potential and voltage applied, respectively.

## 3. Results and Discussion

### 3.1. UV-Vis and XRD Analysis

**Figure 1:**
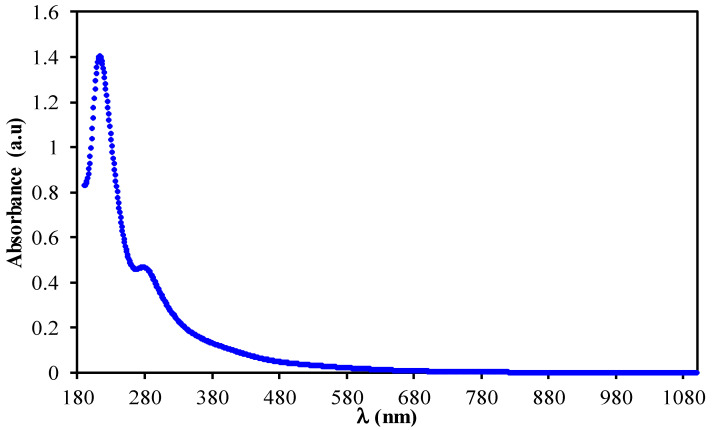
Absorption spectrum for colloidal suspension of Ni^2+^-metal complex.

Green synthesized Ni-metal complex is characterized by UV-Vis absorption spectroscopy, as shown in Figure 1. Since it begins at visible ranges and ends at UV ranges, one can say the absorption spectrum has covered the whole visible range. It is noted that this kind of absorption spectrum can only be observed for semiconducting-based materials [29]. The obtained UV-Vis result of this study is similar to the one for iron–metal complexes reported by Wang et al. [30], which was synthesized by green methodology using various extracts, such as *Rosemarinus officinalis*, *Eucalyptus tereticornis* and *Melaleuca nesophila*. It has been reported that to reveal a surface plasmon resonance (SPR) absorption in the range of UV-visible parts, the size of metal particles must be in the range of nanometer [31]. However, owing to the absence of visible SPR absorption in the Ni^2+^-metal complex (see Figure 1), it cannot be claimed that the Ni^2+^-metal complex exhibited metal characteristics on the particle surfaces due to polyphenols capping. In a previous study, chitosan-based polymer electrolytes have been documented to demonstrate SPR peak in the range of 500 to 800 nm due to copper nanoparticles [32].

**Figure 2:**
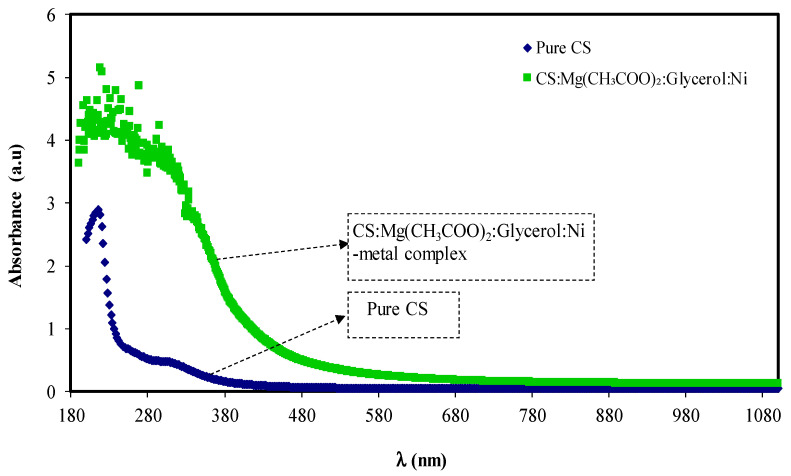
Absorption spectra of pure chitosan (CS) and CS:Mg(CH3COO)2:Gly:Ni composite system.

The absorption spectrums of pure CS and CS:Mg(CH_3_COO)_2_:Gly:Ni composite are shown in Figure 2. The pure CS displays no absorption peak at the visible range, while the Ni-metal complex contained samples that demonstrate distinct absorption from UV to visible ranges. The absorption spectra shift to the visible ranges in the composite samples identifies the Ni-metal complex’s effect on the CS optical properties.

**Figure 3:**
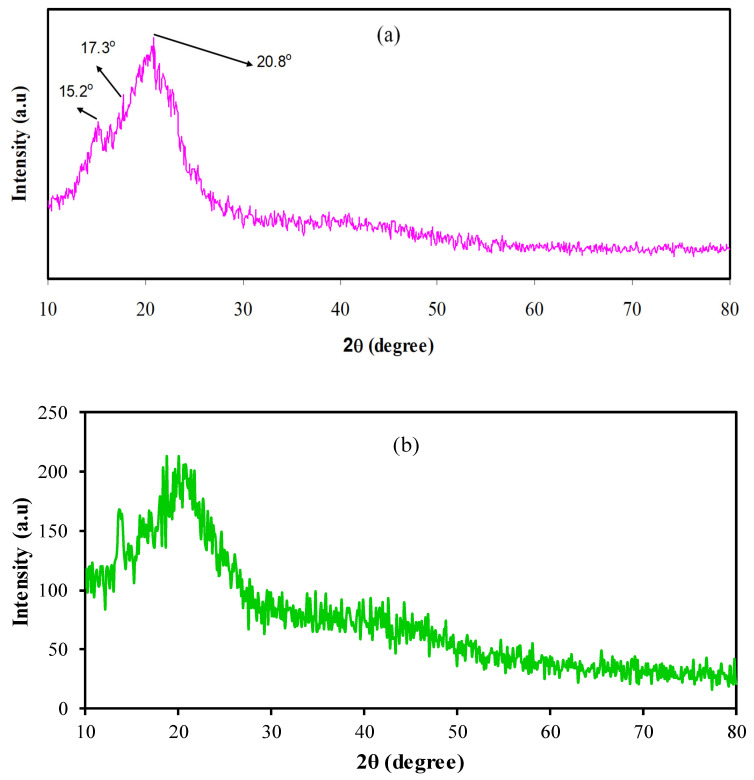
X-ray diffraction (XRD) patterns of (**a**) pure CS (**b**) CS:Mg(CH_3_COO)_2_:Gly:Ni CS systems.

Figure 3a,b show the X-ray diffraction (XRD) pattern for the pure CS and CS:Mg(CH_3_COO)_2_:Gly:Ni films, respectively. Here, two broad peaks at 2θ = 15.1° and 20.9°, with nano crystallite peaks that are too small to yield diffraction peaks can be seen in Figure 3a for the pure CS film. Such broad peaks (i.e., 15.1° and 20.9°) can be attributed to (110) and (220) reflection planes, respectively [33,34]. Previous research has shown that intramolecular and intermolecular hydrogen bonds are mainly responsible for the chitosan’s rigid structure [33]. It indicates the average intermolecular distance of the chitosan’s crystalline parts [35,36]. The XRD pattern of CS:Mg(CH_3_COO)_2_:Gly:Ni complex system is shown in Figure 3b. It is evident from Figure 3b that the nano crystallite peaks of CS have scarified, and only two broad peaks have remained. This reveals that amorphous regions have been enhanced in the CS:Mg(CH_3_COO)_2_:Gly:Ni electrolyte system.

### 3.2. Impedance and Ion Transport Study

To grasp the mechanism of charge transport in PEs, impedance study is fundamentally and technologically crucial. It is essentially important to measure impedance of complex materials for typifying charge transport mechanism [37]. Electrochemical impedance spectroscopy (EIS) is an efficient and informative technique for studying ionic conductivity. 

**Figure 4:**
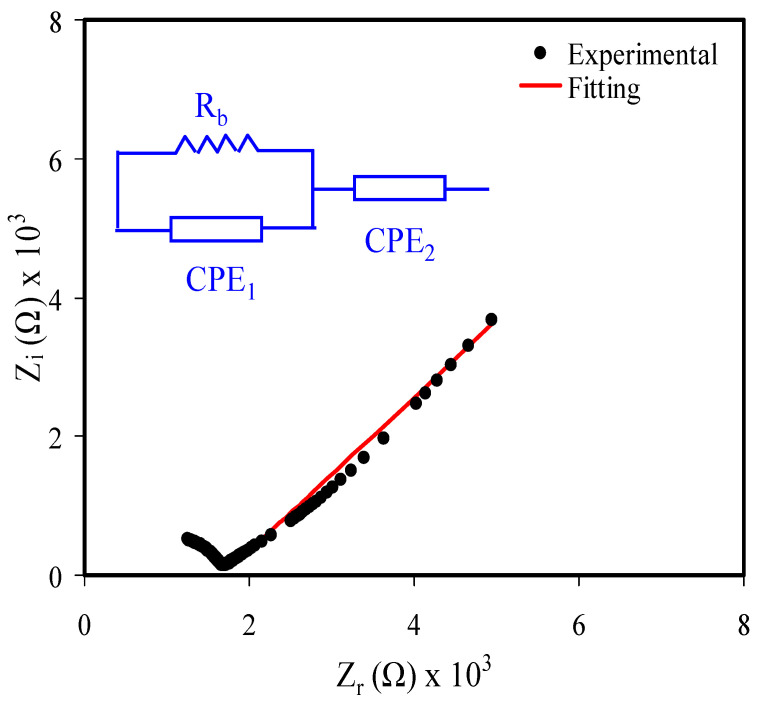
Electrical impedance plot and equivalent circuit diagram (inset) of the prepared polymer electrolyte.

Over the last decade, ion-conducting membrane has drawn the attention of researchers to be utilized as an extensive choice of solid state devices [38]. EIS is regularly employed to distinguish between a relatively small semicircle response at high frequency from stimulus of the ionic conduction in the bulk material and a tail at low frequency as a result of the electrode polarization effect [21,39,40]. Figure 4 shows the impedance spectra and EEC model of the sample. As stated previously, the general profile of the system can be imagined from the fitting of the experimental data points of impedance spectra with the EEC model as exhibited in the inset of Figure 4. At low frequency region, the data point responses arise from the electrode polarization effect as a consequence of the double capacitance layer formation at the interfacial region between the sample and the electrodes. An ideal capacitance is featured from a vertical spike at 90° at low frequency region. However, the case in this study comprises a spike response angled at less than 90° other than the vertical one. This can be ascribed to lack of smoothness of the film or electrode polarization (EP) effect [41,42]. All necessary parameters of the circuit for the prepared sample are accessible in Table 1.

The Z_r_ and Z_i_ components of impedance data are presented in the Nyquist plot and the bulk resistance (R_b_) can be extracted from the cutoff of the plot with the Z_r_ and is important for conductivity calculation following equation [18]:

(11) $$\sigma_{\mathrm{dc}}=\left[\frac{1}{R_{b}}\right]\times \left[\frac{t}{A}\right]$$

where t is the thickness of the film and A is the geometric surface area.

The DC conductivity and transport parameters are listed in Table 2. Generally, the obtained conductivity in the current case is lower compared to the aqueous or ionic liquid-based counterparts. It is strongly preferred to have an ion-conducting electrolyte with high DC ionic conductivity, usually in the range of 10^−5^ to 10^−3^ S/cm, to be commercialized in electrochemical devices. Based on this requirement, the sample that was presented in this work is suitable to be used in the electrochemical devices but it is fundamentally important to modify it in the near future.

### 3.3. Electrochemical Studies

#### 3.3.1. Transference Number Measurement (TNM)

**Figure 5:**
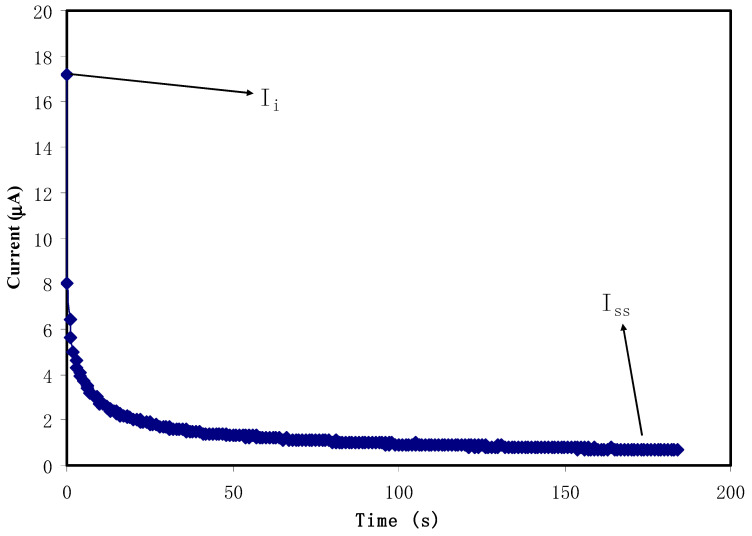
Polarization current against time for the prepared polymer electrolyte sample.

The total conductivity of an electrolyte is from both electrons and ions of the doped salt. In order to confirm the polarization in the electrolyte, TNM analysis has been conducted at 0.8 V. This is one of the important analyses before the fabrication of EDLC. Figure 5 portrays the plot of current against time for the polymer-conducting CS-glycerol-Mg(CH_3_COO)_2_-Ni system. Both contribution of ions and electron can be seen at the initial seconds due to the high current value at 17.2 μA. The ionic blocking mechanism by stainless steel leads to the reduction in current value. At this point, cations and anions from the salt have started to form polarization at negative and positive electrodes, respectively. Complete polarization can be observed as the current value begins to stabilize at 0.7 μA. The high value at the initial seconds, rapid current drop and current stabilization are the indicator of an ionic conductor [19]. The t_ion_ and t_el_ for CS -glycerol-Mg(CH_3_COO)_2_-Ni are 0.96 and 0.04, respectively. The result is comparable to other reported works of polymer electrolytes with various magnesium salts [18,43,44].

#### 3.3.2. Linear Sweep Voltammetry (LSV) Study

**Figure 6:**
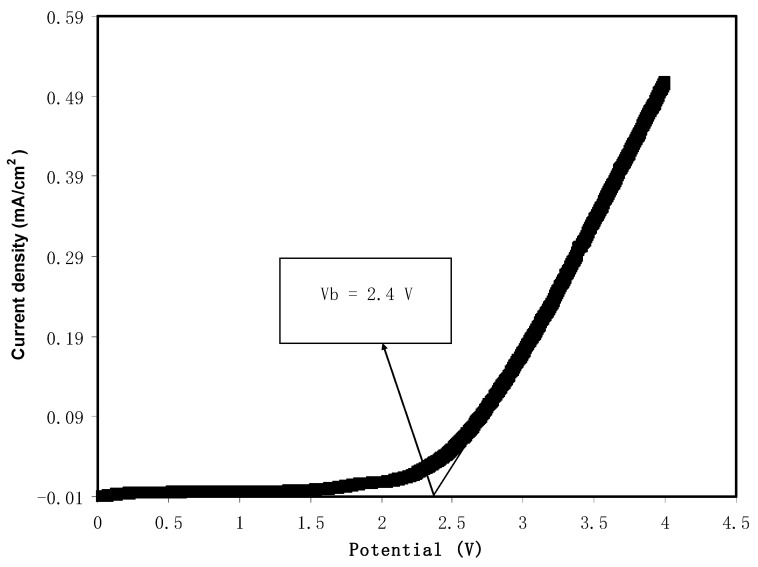
Linear sweep voltammetry (LSV) curve of the prepared polymer electrolyte system.

The breakdown potential of CS-glycerol-Mg(CH_3_COO)_2_-Ni is determined using LSV. It is important to know at which potential the electrolyte will undergo oxidation and start to degrade before the implementation in energy storage devices such as solar cells, batteries and SCs [45]. Figure 6 shows that the electrolyte is stable as there are no current changes observed below potential of 2.4 V. Sharp current increase can be noted beyond 2.4 V. This is due to disruption of polymer–polymer and polymer–salt interaction that leads to polymer degradation [46]. This result shows that the current system has a higher electrochemical potential window compared to many other aqueous electrolytes, while being smaller than the ionic liquid (IL) electrolytes, which are in the range of 3.5 to 4 V [4,10]. Poly(ether urethane)-magnesium perchlorate (Mg(ClO_4_)_2_) has been reported to be stable up to 1.9 V by Jo et al. [47]. In the work of Zainol et al. [48], polymethacrylate (PMMA)-magnesium triflate (Mg(CF_3_SO_3_)_2_) electrolyte has shown that the potential stability is less than 2.5 V. The outcome in this work is analogous to the other magnesium studies, and can be included in the fabrication of EDLC.

### 3.4. EDLC Characterization

**Figure 7:**
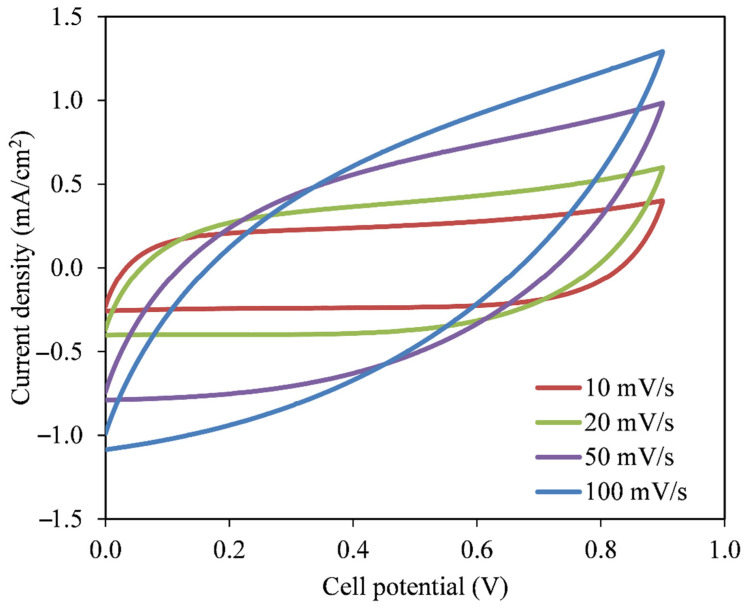
Variation in cyclic voltammetry (CV) plot shapes with changing the scan rate for the fabricated electrical double-layer capacitor (EDLC).

The EDLC assembly with an arrangement of activated carbon | CS -glycerol-Mg(CH_3_COO)_2_-Ni | activated carbon was preliminarily checked using cyclic voltammetry (CV). It is well known that the capacitor is scan-rate-dependent, thus various scan rates from 10 to 100 mV/s were used. The shape of CV plot in Figure 7 varies from rectangular to leaf-like shapes without any obvious peaks. Jäckel et al. [49] stated that a peak in CV plot usually signifies the presence of Faradaic current due to intercalation and deintercalation, which does not happen in a capacitor. At large scan rate, the transportation of mobile ions occurs at a rapid rate. In addition to that, due to internal resistance and carbon porosity, current dependence of voltage is created [50]. Table 3 shows the capacitance value of the EDLC. It is obvious that C_cv_ is large at low scan rate and vice versa. At low scan rate, as expected, the response of CV is relatively plateau, indicating ion accumulation at the electrode|electrolyte boundary with low ohmic resistance as shown in Figure 7 [51,52].

**Figure 8:**
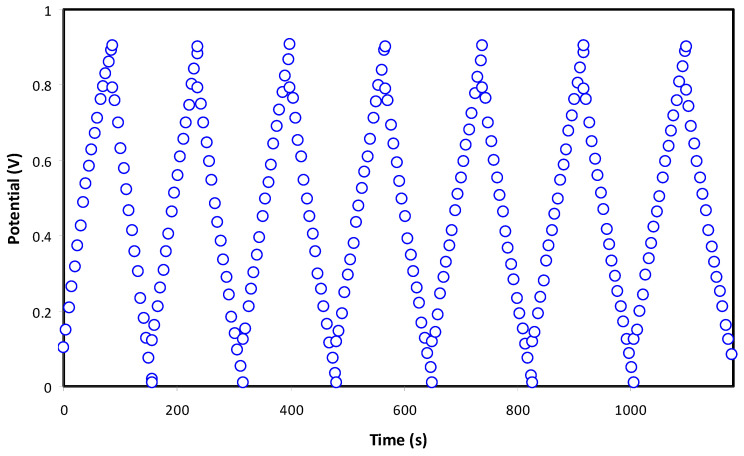
Charge–discharge pattern of the fabricated electric double layer capacitor (EDLC) at specific cycles.

**Figure 9:**
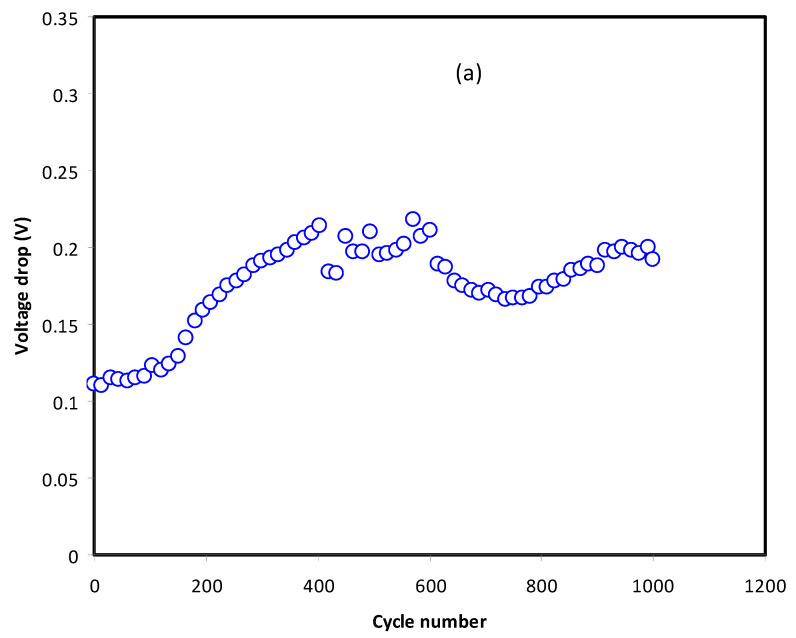

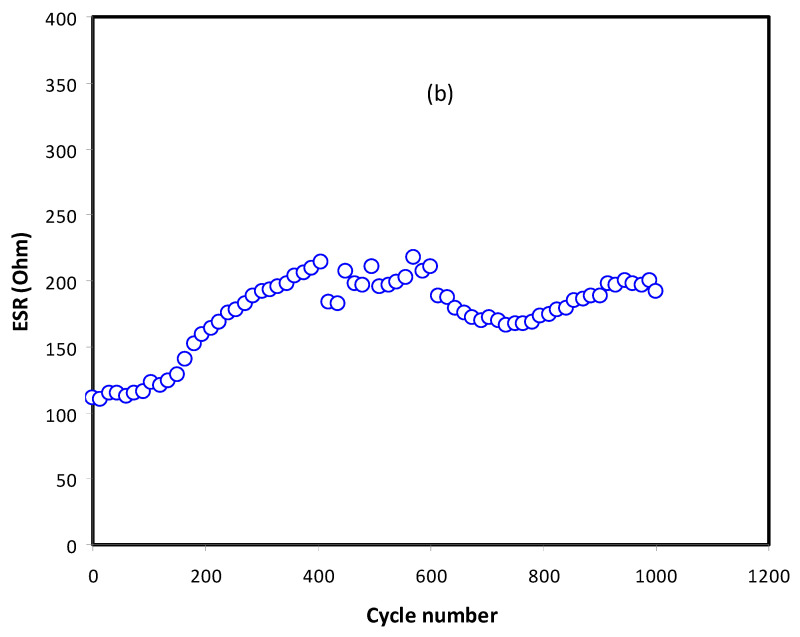
(**a**) Voltage drop pattern and (**b**) equivalent series resistance (ESR) pattern of the EDLC throughout the 1000 cycles.

A good capacitor will portray a triangle shape charge–discharge plot as in Figure 8. The linearity of both the charging and discharging process is an indicator of the polarization process; whereas, it is non-linear for conventional batteries [53]. Capacitance stands for the ratio of electrical charge changes in a system corresponding to changes in the potential. A small potential drop before discharge process is common in an EDLC due to the existence of internal resistance. Figure 9a shows the voltage drop, while Figure 9b shows the ESR of the EDLC. The voltage drops at cycles less than 200 in the range of 0.14 V. Beyond 200 cycles, voltage drop started to increase as the polarization process is obstructed by the increment in ESR as shown in Figure 9b. At high cycles, number in a rapid charge–discharge leads some ions to undergo recombination to form neutral ion pairs. Ion pairs can lead to improper polarization built at the surface of the electrodes [54]. However, the values of voltage drop and ESR started to stabilize from 400th to 1000th cycles. At some point of charge–discharge, cations and anions achieved a stable double layer process, which leads to stable internal resistance. 

**Figure 10:**
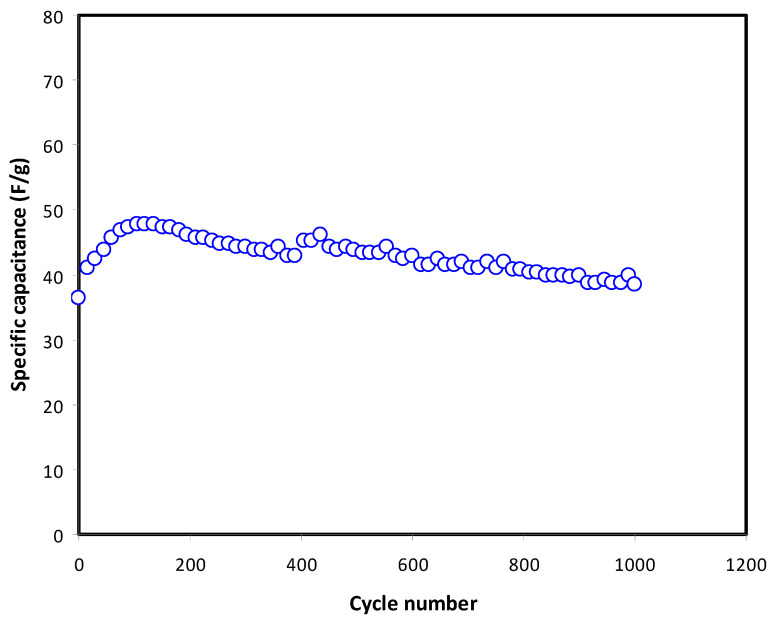
Satiability in specific capacitance value of the fabricated EDLC up to 1000 cycles at 0.5 mA/cm^2^.

Figure 10 illustrates the C_CD_ of the EDLC throughout the 1000 cycles. The value of the *C_CD_* is observed to increase from 36.4 F/g (1st cycle) to 47.9 F/g (135th cycle) and drops to 45.2 F/g (400th cycle). The performance of the EDLC is typically unstable before stabilization occurs. This usually happens since the ions are still trying to recognize the patterns of the polarization at the initial stage. As the cycle number exceeds 400, *C_CD_* value becomes constant up to 1000 cycles with an average of 41.7 F/g. As reported in our previous work, CS-glycerol-Mg(CH_3_COO)_2_ can only be charged and discharged up to 400 cycles [23]; while, in this work CS-glycerol-Mg(CH_3_COO)-Ni reaches up to 1000 cycles. This could be the effect of the addition of metal complexes. Metal complexes (Fe, Ce, Cu, Zn and Ni) have good electrical conduction properties as well as large surface area, which is beneficial for electrochemical devices [55,56,57]. The conduction of ion is usually preferable in amorphous region of the electrolyte. The increase in cycle number due to the addition of Ni could be related to the improvement of amorphousness of the electrolyte. Brza et al. [24], reported that the addition of Cu(II) to polyvinyl alcohol (PVA) has improved the amorphous nature of the electrolyte. This is caused by the interaction of metal complexes with polymers, which reduce the crystallinity.

**Figure 11:**
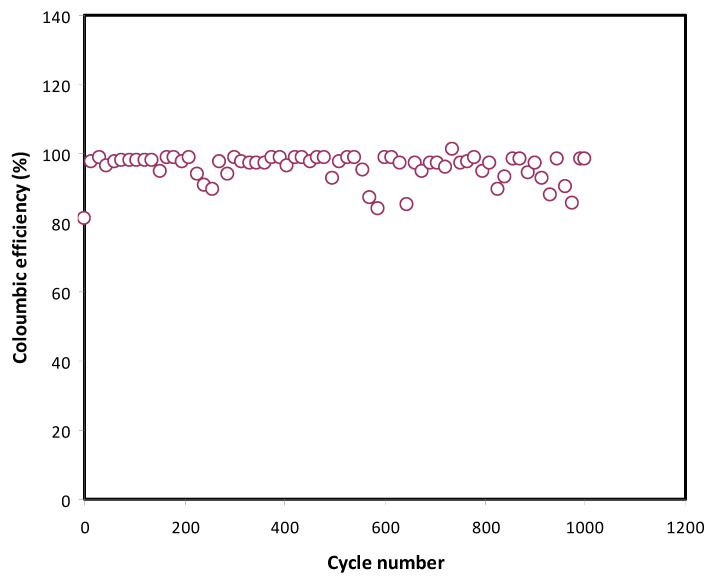
Coulombic efficiency plot over 1000 cycle for the fabricated EDLC.

The cycle stability of the EDLC can be detected from the consistent value of efficiency. Apart from being consistent, efficiency of an excellent EDLC is large, which is more than 95%. The efficiency was calculated using:

(12) $$\mathrm{Efficiency}=\frac{t_{\mathrm{dis}}}{t_{\mathrm{cha}}}$$

where t_dis_ and t_cha_ are time for one full discharging–charging, respectively. Efficiency of the fabricated EDLC is plotted in Figure 11. An efficiency of 81.2% can be seen at the 1st cycle and it increases to 97.5% at the 15th cycle. Normally, at initial cycle numbers, the charging process is longer than discharging. Ions start to migrate towards the surface of the electrode that is completely different from that of desorbing from the electrode surface. At the beginning, the system needs time to reach stabilization and shows relatively low efficiency. Beyond the 15th cycle, the efficiency tends to stabilize with an average value of 96.1% up to 1000 cycles. It is proven that the discharging process lasts a longer time than the charging process, indicating high efficiency. In this work, the results indicate that the EDLC assembly possesses excellent stability and electrolyte–electrode compatibility.

**Figure 12:**
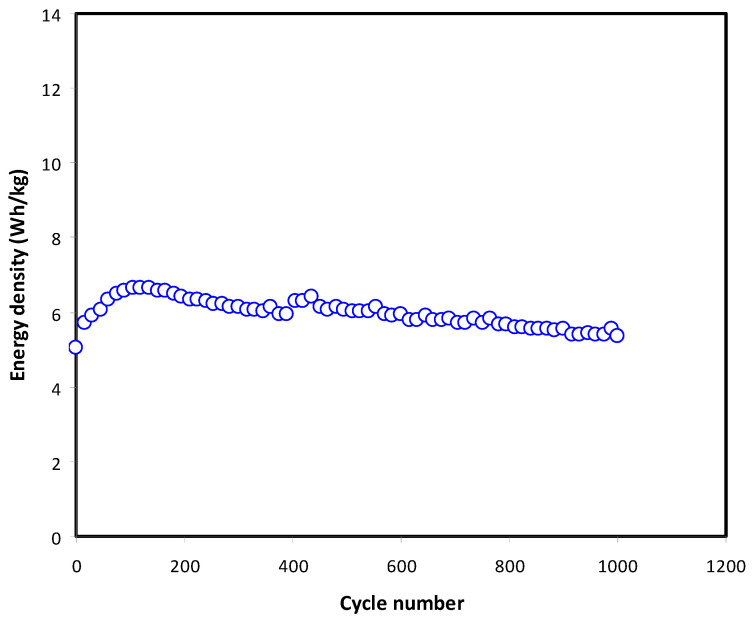
The plot of energy (*E*) of the fabricated EDLC throughout 1000 cycles.

The energy storing quantity in a given system is called energy density. Figure 12 exhibits the trend of energy density variation against cycle number over 1000 cycles. It is noted that the trend of *E* is comparable to *C_CD_* in which the performance suffers from fluctuation at cycles below 200 as shown in Figure 10. It is intuitive that energy density is in direct proportionality to specific capacitance. Beyond cycle number of 200, stabilization of *E* can be clearly seen up to 1000 with an average value of ~5.86 Wh kg^−1^. This phenomenon can be explained on the basis of the fact that in this system, ions can pass omitting energy barrier for each charging and discharging cycle during the transportation process [58]. Winie et al. [59] recorded an EDLC assembly using a CS host that possesses energy density lying from 0.57 to 2.8 Wh/kg by taking the current density from 2 to 0.6 mA/cm^2^, respectively. As stated by Bandaranayake et al. [60], polyacrylonitrile-magnesium chloride (MgCl_2_)-based EDLC provided an energy density of 5 Wh/kg. A consistent trend of capacitance and energy density indicate that ions recombination process is less dominant. 

**Figure 13:**
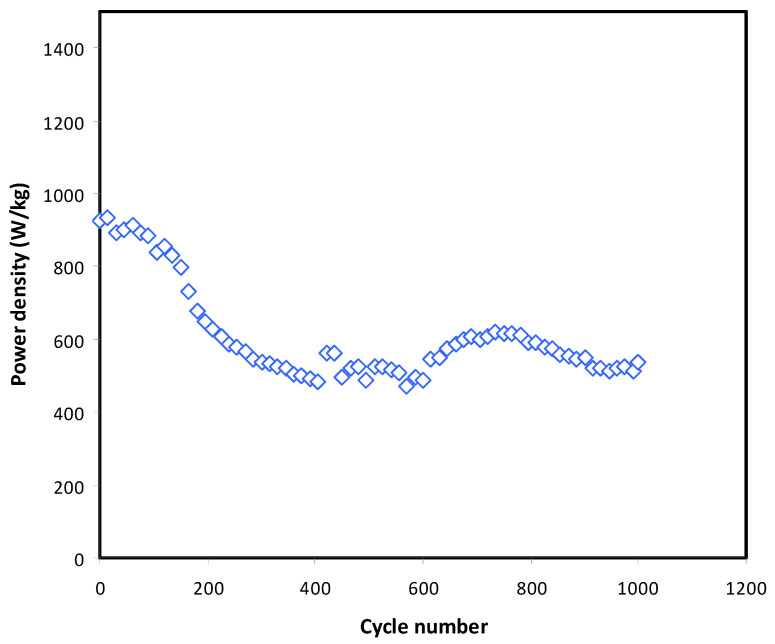
Power density of the fabricated EDLC throughout 1000 cycles.

One advantage of SCs that conventional batteries do not have is high power density. This is because cations and anions in batteries require more energy to be discharged or charged as the ions have to deintercalate out of the electrolyte. Figure 13 shows the power delivered by the EDLC at 0.5 mA/cm^2^. A steep gradient of *P* reduction from 1st (924 W/kg) to 200th (628.0 W/kg) can be explained by the increased internal resistance in the EDLC. ESR of the EDLC increased from 111 ohm to 163 ohm at 200th cycles. Power delivered by the EDLC is observed to be more stable beyond 200th cycles. As stated in Yassine and Drazen [61], power density is strongly related to the ESR of the EDLC. High ESR means it is hard for ions to be adsorbed, thus delivering lower power. The plasticized polymer electrolyte with Ni metal complexes in this study has presented a significant improvement in the overall performance of the fabricated EDLC. Generally, gel polymer electrolytes (GPEs) show enhanced ambient conductivity; however, they suffer from reduced mechanical integrity of the film as well as increased corrosive reactivity of polymer electrolyte towards the metal electrode [62,63]. The current studied polymer electrolyte system has shown an effective way to minimize these drawbacks through introducing metal complex and using adequate amount of plasticizer. The employed metal complex undergoes interaction with the host polymers chains, which in turn reduce the crystallinity and enhance conductivity. Thus, metal complex can be introduced to reduce the amount of used plasticizer and thereby lessen the interaction of electrolyte with metal electrodes, which results in more stability and longer life-cycle [62,63]. 

The outcomes of this work highlighted the role of both plasticizer and metal complexes in improving the properties of the polymer electrolytes. Thus, these approaches can be considered as effective methods to enhance the properties of the polymer-based electrolytes in order to be suitable for commercialization and meet the industrial level. Table 4 presents the general performance of the fabricated EDLC compared to many other polymer-based and gel electrolyte systems from the literature. 

## 4. Conclusions

The performance of CS-Mg(CH_3_COO)_2_ system has been enhanced with the presence of glycerol and Nickel (Ni) metal complexes. The optical study highlighted a clear shift in absorption spectra to the visible ranges with the addition of Ni metal complexes. The XRD patterns of the Ni metal complex doped sample have shown diminishing of many crystalline peaks of pure CS, which confirmed the impact of Ni metal complexes on enhancing the amorphous nature of the sample. Through the simulation of impedance data, a variety of ion transport parameters were calculated. The DC conductivity and other transport parameters point to the appropriateness of the sample for EDLC application. The overall conductivity has been determined by TNM technique to be predominantly contributed to by ions rather than electron species. High ionic transference number proves this statement. The electrolyte has a potential stability up to 2.4 V as the oxidation current increases beyond that potential. EDLC in this work is scan-rate-dependent and high capacitance value is obtained at low scan rate. The performances of the EDLC are unstable at cycle less than 200th. However, stabilization is achieved beyond that. The fabricated EDLC device was found to be safe up to 1000 cycles without possessing great breakdown voltage. Thus, the stabilization of the EDLC performance could be improved via the dispersion of metal complexes.

## Figures and Tables

**Table 1:** Various parameters of the circuit elements for the prepared polymer electrolyte system.

Electrical Equivalent Circuit (EEC) Parameters	Values
n_1_ (rad)	0.71
n_2_ (rad)	0.53
K_1_ (F^−1^)	2.6 × 10^8^
K_2_ (F^−1^)	1.05 × 10^5^
Y_1_ (F)	3.85 × 10^−9^
Y_2_ (F)	9.52 × 10^−6^
R_b_ (Ω)	1.6 × 10^3^

**Table 2:** Ion transport parameters of the prepared polymer electrolyte system.

Ion Transport Parameters	Values
σ_dc_ (S cm^−1^)	1.09 × 10^−5^
D (cm^2^ s^−1^)	4.91 × 10^−8^
µ (cm^2^ V^−1^ s)	1.91 × 10^−6^
n (cm^−3^)	3.55 × 10^19^

**Table 3:** Variation in capacitance values with respect to different scan rates.

Scan Rate (mV/s)	Capacitance (F/g)
10	27.793
20	19.835
50	11.349
100	6.032

**Table 4:** General performance of the fabricated EDLC device compared to the other EDLC devices based on various polymer electrolytes in terms of specific capacitance (C_s_), energy density (E), power density (P) and cycle number.

Electrolyte System	C_s_ (F g^−1^)	E (Wh kg^−1^)	P (W kg^−1^)	Cycle No.	Ref.
Dextran:NH_4_Br	2.05	-	-	100	[64]
PVA:Dextran:NH_4_I	4.2	0.55	64	100	[65]
Corn starch: LiClO_4_: SiO_2_	9.83	0.9	135	500	[66]
CS:MC:NH_4_I:Gly	9.97	1.1	578.55	100	[67]
PVA:LiClO_4_:TiO_2_	12.5	1.56	198.7	1000	[68]
CS-κ-carrageenan-NH_4_NO_3_	18.5	-	1.8	20	[69]
PVA:CH_3_COONH_4_:BmImBr	21.89	1.36	34.66	500	[70]
CS-PVA-NH_4_NO_3_-EC	27.1	-	-	100	[71]
MC:PS:NH_4_NO_3_:Gly	31	2.3	385	1000	[72]
CS-PVA-Mg(CF_3_SO_3_)_2_:GL	32.69	-		100	[73]
MC-NH_4_NO_3_- PEG	38	3.9	140	100	[74]
**CS:Mg(CH_3_COO)_2_:Gly:Ni**	**41.7**	**5.86**	**628**	**1000**	**This work**

Where: NH_4_Br = ammonium bromide, MC = methylcellulose, NH_4_I = ammonium iodide, LiClO_4_ = lithium perchlorate, SiO_2_ = silicon dioxide, TiO_2_ = titanium dioxide, NH_4_NO_3_ = ammonium nitrate, CH_3_COONH_4_ = ammonium acetate, BmImBr = 1-butyl-3-methylimidazolium bromide, EC = ethylene glycol, PS = potato starch, PEG = poly(ethylene glycol), Mg(CF_3_SO_3_)_2_ = magnesium triflate.

## Data Availability

Exclude this statement because the study did not report any data.
